# Biochemistry, genetics and biotechnology of glycerol utilization in *Pseudomonas* species

**DOI:** 10.1111/1751-7915.13400

**Published:** 2019-03-18

**Authors:** Ignacio Poblete‐Castro, Christoph Wittmann, Pablo I. Nikel

**Affiliations:** ^1^ Biosystems Engineering Laboratory Center for Bioinformatics and Integrative Biology Faculty of Natural Sciences Universidad Andrés Bello Santiago de Chile Chile; ^2^ Institute of Systems Biotechnology Universität des Saarlandes Saarbrücken Germany; ^3^ The Novo Nordisk Foundation Center for Biosustainability Technical University of Denmark Kgs Lyngby Denmark

## Abstract

The use of renewable waste feedstocks is an environment‐friendly choice contributing to the reduction of waste treatment costs and increasing the economic value of industrial by‐products. Glycerol (1,2,3‐propanetriol), a simple polyol compound widely distributed in biological systems, constitutes a prime example of a relatively cheap and readily available substrate to be used in bioprocesses. Extensively exploited as an ingredient in the food and pharmaceutical industries, glycerol is also the main by‐product of biodiesel production, which has resulted in a progressive drop in substrate price over the years. Consequently, glycerol has become an attractive substrate in biotechnology, and several chemical commodities currently produced from petroleum have been shown to be obtained from this polyol using whole‐cell biocatalysts with both wild‐type and engineered bacterial strains. *Pseudomonas* species, endowed with a versatile and rich metabolism, have been adopted for the conversion of glycerol into value‐added products (ranging from simple molecules to structurally complex biopolymers, e.g. polyhydroxyalkanoates), and a number of metabolic engineering strategies have been deployed to increase the number of applications of glycerol as a cost‐effective substrate. The unique genetic and metabolic features of glycerol‐grown *Pseudomonas* are presented in this review, along with relevant examples of bioprocesses based on this substrate – and the synthetic biology and metabolic engineering strategies implemented in bacteria of this genus aimed at glycerol valorization.

## Graphical abstract

Glycerol is a relevant substrate for biotechnology. Yet, the metabolic, physiological, and genetic features of its use has been unveiled only for a few microbial species. In this review article, the use of glycerol as a substrate for Pseudomonas species is described by focusing on the genetic, physiology and metabolic traits of cells grown on the polyol.

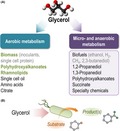

## Introduction

Contemporary synthetic biology and metabolic engineering offer the possibility of expanding the substrate range of microbial cell factories beyond the sugars typically used as carbon sources (Calero and Nikel, 2019; Prather, 2019). Examples of this sort of metabolic manipulation for broadening substrate ‘palatability’ of bacteria include several chemical species, ranging from simple C1 compounds such as CO_2_ or HCOOH (Antonovsky *et* *al.,* 2016; Yishai *et* *al.,* 2018) to structurally complex substrates such as lignocellulosic materials derived from biomass (Beckham *et* *al.,* 2016; Barton *et* *al.,* 2018; Kim and Woo, 2018). Alcohols conform a special category of alternative substrates for biotechnology, and they are currently being discussed as promising renewables for sustainable bioproduction (Stowell *et* *al.,* 1987; Smith, 2004; Dahod *et* *al.,* 2010; Hoffmann *et* *al.,* 2018). Glycerol (1,2,3‐propanetriol, C_3_H_8_O_3_), for instance, is a widely available, versatile and structurally simple compound that can be used as a carbon source or as a precursor in a variety of chemical and biological conversions. This polyol has been traditionally used in multiple industrially relevant areas, e.g. as an ingredient in foods and beverages (by exploiting its sweetening properties; in fact, the name *glycerol* is derived from the Greek γλυκερός, ‘sweet’), as well as pharmaceuticals and cosmetic products, both as solvent and humectant (Pagliaro and Rossi *et* *al.,* 2008b).

Biodiesel is a fuel comprised of monoalkyl (methyl, ethyl or propyl) esters of long‐chain fatty acids derived from vegetable oils or animal fats (Hollinshead *et* *al.,* 2014). Its value as a fuel has been recognized as early as the 19^th^ century: the transesterification of a vegetable oil catalysed by a base was conducted four decades before the first diesel engine became functional (Henriques, 1898). Biodiesel has promising lubricating properties and cetane ratings compared to low sulfur diesel fuels, with a calorific value of about 37 MJ kg^−1^. The current transesterification process used for biodiesel production involves the treatment of yellow grease (recycled vegetable oil), virgin vegetable oil or tallow with a mixture of NaOH or KOH and CH_3_OH (van Gerpen and Knothe, 2010). The main by‐product of this production process is glycerol: ca. 10 kg of crude glycerol is generated for every 100 kg of biodiesel produced. The fast development of the biofuel industry in several countries over the last three decades (with a global production volume of 3.8 million tons in 2005) has generated a considerable amount of crude glycerol (Suppes, 2010). Approximately 85% of all the biodiesel production over the last decade came from the European Union (Ntziachristos *et* *al.,* 2014). In addition, the bioethanol process (using *Saccharomyces cerevisiae* as biocatalyst) generates glycerol up to 10% of the total sugar (usually sucrose) consumed in the fermentation (Hasunuma and Kondo, 2012; Mohd Azhar *et* *al.,* 2017). As a consequence of this global situation, the last 10 years have witnessed the rise of glycerol as a very attractive substrate for bacterial fermentations (Mota *et* *al.,* 2017). The excess of crude glycerol produced in the biofuel industry led to a decrease in glycerol price, and some years ago, it was even considered a waste (with an associated disposal cost) by many biodiesel‐production plants. Converting crude glycerol into value‐added products thus became a relevant need to improve the viability of the biofuel economy (Pagliaro and Rossi *et* *al.,* 2008a), and both chemical and biological approaches have been explored to convert glycerol into more valuable products. Considering that crude glycerol is a non‐edible renewable, its use has also advantages in terms of sustainability as it does not compete with other substrates that could be otherwise used in the food industry (Stichnothe, 2019). Compared to chemical routes for transformation of the polyol, biological transformation offers several advantages, ranging from less energy use (thus making the process more environment‐friendly) to higher specificity, and increased tolerance to impurities such as salts and CH_3_OH, both of which occur at high levels in crude glycerol (Katryniok *et* *al.,* 2009). Over the last few years, however, global markets have changed and oil prices have stabilized – which has directly impacted biodiesel production (Pagliaro, 2017). Nevertheless, glycerol continues to attract attention as a substrate for biotechnology as it can be used by a myriad of microorganisms for the synthesis of a wide range of bioproducts (da Silva *et* *al.,* 2009; Dobson *et* *al.,* 2012; Pettinari *et* *al.,* 2012; Mattam *et* *al.,* 2013; Mitrea *et* *al.,* 2017). Moreover, current trends indicate that biodiesel will become the clean liquid fuel of choice in many countries, especially in those that have legal requirements to use alternatives to petrochemical fuels (Guo and Song, 2019). The United States Environmental Protection Agency, for instance, established a fuel standard volume requirement for biodiesel of 8 millon litres for 2019 (Weaver, 2018) – requirements that will inevitably result in an increasing availability of raw glycerol.

Interestingly, the biotechnological value of glycerol as a substrate has been recognized since the early times of industrial microbiology (Johnson, 1947; Gunsalus *et* *al.,* 1955). In fact, some of the oldest examples of technical‐scale bioreactor fermentations include the transformation of glycerol into biomass and reduced biochemical products. Nakas *et* *al*. (1983), for instance, described the fermentation of glycerol by *Clostridium pasteurianum* in an attempt to obtain a marketable product [a mixture of *n*‐butanol, 1,3‐propanediol (1,3‐PDO) and ethanol] from glycerol photosynthetically formed by halophilic *Dunaliella* algae. Due to the more reduced nature of the carbon atoms in glycerol as compared to sugars (e.g. glucose and xylose, customary substrates in bioprocesses), the polyol is mostly processed *via* oxidative metabolism in aerobic processes. There are, however, several bacteria that can ferment this substrate anoxically, e.g. some clostridia and a few enterobacteria (Hatti‐Kaul and Mattiasson, 2016) – a circumstance that has been also exploited for the design of industrial bioprocesses. Until the last decade, for instance, it was widely accepted that *Escherichia coli* was unable to use glycerol as a substrate in the absence of external electron acceptors (Booth, 2005). Since then, several studies describing the fermentation of glycerol by different wild‐type or mutant *E*. *coli* strains have paved the way for the efficient use of this low‐cost, readily available substrate to synthesize a variety of biotechnologically relevant products under different oxygen availability conditions (Yazdani and González, 2007; Murarka *et* *al.,* 2008; Nikel *et al*., 2006, 2008a, 2010a) – thus increasing the sustainability of fermentation processes using this polyol as the substrate. The higher degree of reduction of glycerol (γ = 4.7) over glucose (γ = 4) facilitates the synthesis of reduced bioproducts as demonstrated in *E*. *coli* strains (Nikel *et al.,* 2008a,2008b, 2010b). Since less carbon has to be oxidized into CO_2_ to generate reducing power, the use of glycerol potentially offers higher yields on substrate than when using sugars. Yet, what are the biotechnological uses of glycerol beyond the so‐called model bacterial species?

The last decade has witnessed an exponential increase in the number of studies exploiting *Pseudomonas* species as biocatalysts. In particular, *P*. *putida* KT2440, a non‐pathogenic soil bacterium that has been adapted to laboratory conditions (Nelson *et* *al.,* 2002; Belda *et* *al.,* 2016), has emerged as the *chassis* of choice for engineering biochemical pathways while exploiting its intrinsically high tolerance to different types of physicochemical stresses (Poblete‐Castro *et* *al.,* 2012a, 2017; Nikel *et* *al.,* 2014a,2014b; Nikel and de Lorenzo, 2018a,2018b; Abram and Udaondo, 2019). Several studies have described the use of glycerol by *Pseudomonas* species, and biochemical and genetic studies have disclosed a rather different metabolic operation, genetic regulation and physiological responses as compared to other bacteria. Against this background, in this article, we review our current knowledge on the use of glycerol by *Pseudomonas* species either *via* natural or engineered pathways – with an emphasis on the physiology and metabolism of *P*. *putida* and the many opportunities that this substrate brings forth for biotechnological applications.

## Biochemistry and genetics of glycerol utilization by *Pseudomonas*

### General aspects of glycerol assimilation in bacteria

**Figure 1:**
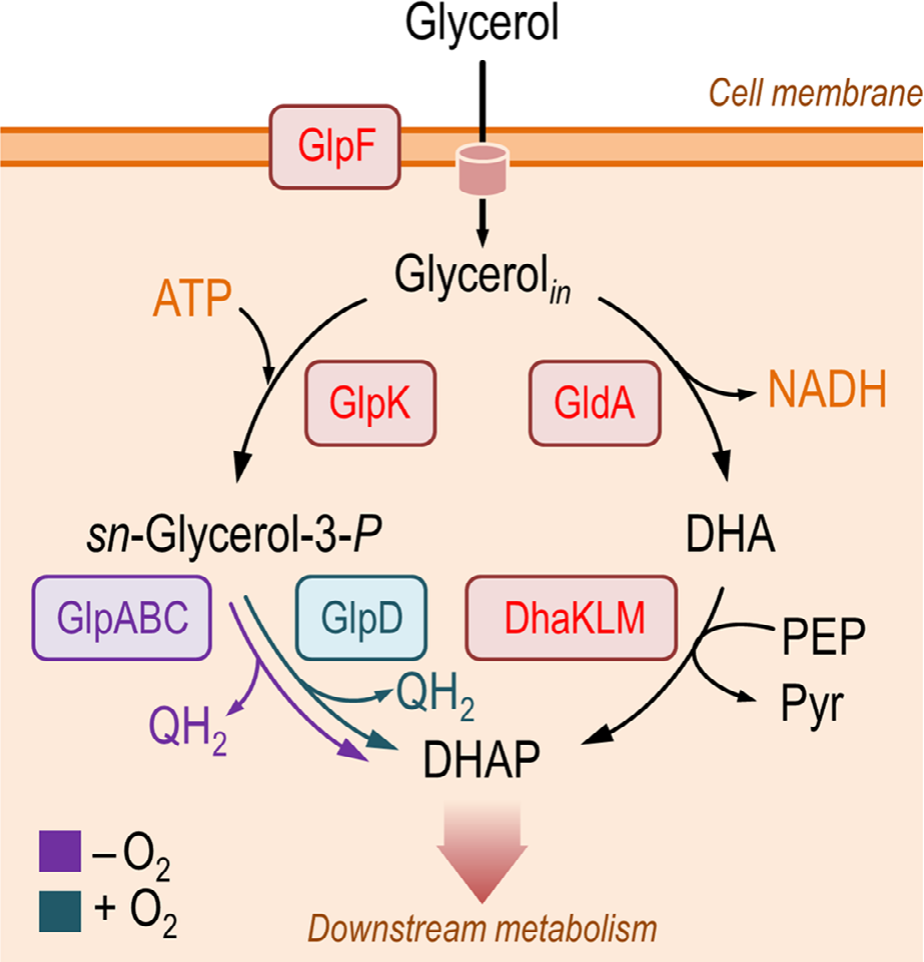
Conserved pathways for glycerol metabolism in bacteria. In most Gram‐negative bacteria, such as *E*. *coli*, alternative catabolic pathways ultimately lead to the generation of dihydroxyacetone‐*P* (DHAP), which is later channelled into key glycolytic intermediates *via* downstream metabolism. Apart from the direct, ATP‐dependent phosphorylation of intracellular glycerol (glycerol_*in*_) indicated to the *left*, the polyol can be oxidized into dihydroxyacetone (DHA), and then phosphorylated using phosphoenolpyruvate (PEP) as the phosphoryl donor (as shown to the *right*), thereby generating pyruvate (Pyr). The enzymes involved in glycerol metabolism are GlpF, glycerol facilitator (transporter); GlpK, glycerol kinase; GlpABC, (anaerobic) *sn*‐glycerol‐3‐*P* dehydrogenase; GlpD, (aerobic) *sn*‐glycerol‐3‐*P* dehydrogenase; GldA, glycerol dehydrogenase; and DhaKLM, DHA kinase. QH_2_ denotes a reduced quinone (e.g. ubiquinone or menaquinone), which serves a cofactor for a flavin‐containing enzyme. Enzymatic steps indicated in red are independent of the presence of oxygen, whereas the two possible *sn*‐glycerol‐3‐*P* dehydrogenation reactions are identified with different colours depending on the availability of (alternative) electron acceptors.

In a comprehensive review of glycerol metabolism, Lin (1976) had described assimilation pathways present in several bacterial species, with a special focus on *E*. *coli* and related Enterobacteriaceae. Although glycerol processing in bacteria can essentially follow only two possible biochemical routes, the reduced nature of its carbon atoms renders catabolism of this substrate difficult in the absence of external electron acceptors (${\text{NO}}_{3}^{-}$ or fumarate). Irrespective of the pathway followed, phosphorylation and dehydrogenation steps ultimately convert glycerol into dihydroxyacetone‐*P* (DHAP), either aerobically or anaerobically (Fig. 1). DHAP is incorporated into the central carbon metabolism as a key precursor that is further processed by the same glycolytic routes deployed when bacteria grow on sugars. Apart from the direct incorporation of glycerol‐derived metabolites into biomass, this compound can be also converted into a series of reduced by‐products to meet the redox and carbon balance. Bouvet *et* *al*. (1995) described bacterial species, belonging to the genera *Citrobacter*,* Enterobacter* and *Klebsiella*, capable of fermenting glycerol. In these species, there is a reductive pathway for glycerol utilization, in which the substrate is firstly dehydrated by a vitamin B_12_‐dependent enzyme to form 3‐hydroxypropionaldehyde that is further reduced to 1,3‐PDO by an NADH‐linked oxidoreductase (1,3‐PDO dehydrogenase), thereby regenerating NAD^+^. The fermentation of glycerol with the concomitant formation of 1,3‐PDO [and, in some cases, 1,2‐propanediol (1,2‐PDO)] was also described in *Lactobacillus* and *Clostridium* species (Biebl *et* *al.,* 1999).

Apart from passive diffusion, glycerol uptake in bacteria is mediated by glycerol diffusion facilitators, integral membrane proteins catalysing the rapid equilibration of glycerol concentration gradients across the cytoplasmic membrane (Stroud *et* *al.,* 2003). These facilitators are α‐type channels that enable the diffusion of small polyols and related molecules into the cell, and these channels are known because of their exquisite substrate selectivity. They do not permit the passage of charged compounds through them, a feature essential for the maintenance of the electrochemical gradient across the membrane. Once transported, intracellular glycerol is converted to *sn*‐glycerol‐3‐*P* by glycerol kinase (GlpK) using ATP as the phosphoryl donor. The glycerol diffusion facilitator does not recognize *sn*‐glycerol‐3‐*P* as a substrate and this intermediate remains inside the cell, where it is further metabolized. The driving force for the uptake of glycerol is thus generated by substrate phosphorylation by GlpK (Voegele *et* *al.,* 1993). While *sn*‐glycerol‐3‐*P* cannot leave the cytoplasm, it can be imported into the cell by the GlpT transporter, a member of the major facilitator superfamily that couples the import of *sn*‐glycerol‐3‐*P* into the cytoplasm to the export of inorganic phosphates from the cytoplasm to the periplasm (Lemieux *et* *al.,* 2004). In *E*. *coli*,* sn*‐glycerol‐3‐*P* can be further metabolized to DHAP by either of two membrane‐bound enzymes, depending on the growth conditions (Fig. 1). Under aerobic conditions, a homodimeric aerobic *sn*‐glycerol‐3‐*P* dehydrogenase (encoded by *glpD*) is produced, which can accept either oxygen or ${\text{NO}}_{3}^{-}$ as the electron acceptor (Schryvers *et* *al.,* 1978; Yeh *et* *al.,* 2008). Under anaerobic conditions, a different *sn*‐glycerol‐3‐*P* dehydrogenase is preferentially expressed – this tri‐heteromeric protein complex, which is encoded by the *glpABC* operon, channels the electrons from *sn*‐glycerol‐3‐*P* to either ${\text{NO}}_{3}^{-}$ or fumarate (*via* the quinone pool) since oxygen can no longer be used as an electron acceptor (Cole *et* *al.,* 1988). Apart from the obvious role in substrate catabolism, the presumed significance of this process is the salvage of glycerol and glycerol phosphates generated by the breakdown of phospholipids and triacylglycerol (Blom *et* *al.,* 2011).

Apart from these main biochemical reactions, the first to be discovered and collectively known as the *glycerol and glycerophospholipid degradation pathway*,* E*. *coli* K‐12 possesses an NAD^+^‐linked dehydrogenase, termed GldA, which is able to support glycerol fermentation (Fig. 1). Gonzalez *et* *al*. (2008) demonstrated that GldA, annotated as a dual L‐1,2‐PDO dehydrogenase/glycerol dehydrogenase, is involved in glycerol fermentation both as a glycerol dehydrogenase (i.e. generating dihydroxyacetone), and as a 1,2‐PDO dehydrogenase, in this case regenerating NAD^+^ by producing 1,2‐PDO from hydroxyacetone. GldA is also involved in methylglyoxal detoxification (Ko *et* *al.,* 2005). In this branch of glycerol metabolism, dihydroxyacetone is phosphorylated into DHAP by DhaKLM, which uses phosphoenolpyruvate (instead of ATP) as the phosphoryl donor (Jin and Lin, 1984).

### Metabolism of glycerol in *Pseudomonas* species: substrate transport, trunk and auxiliary metabolic pathways

Although the glycerol metabolism indicated in the previous section prevails in most Gram‐negative species (especially in Enterobacteria), members of the *Pseudomonas* genus display relevant differences both in terms of the biochemical and genetic architecture of glycerol utilization. *Pseudomonas* species possess over 300 known and putative nutrient uptake systems, which enable them to metabolize a large number of organic compounds and inhabit many diverse ecological niches (Silby *et* *al.,* 2011). The outer membrane of these bacteria acts as a semi‐permeable barrier – excluding many classes of potentially toxic molecules from the cell. Nutrients use specialized water‐filled channels called *porins* to traverse this physical barrier (Chevalier *et* *al.,* 2017); the actual entry into the *Pseudomonas* cell is mediated by one of four classes of cytoplasmic membrane transporters as follows: glycerol/water facilitators, phosphotransferase systems, primary active transporters and secondary active transporters (Tamber and Hancock, 2003). The first GlpF transporter to be identified in a *Pseudomonas* species was described in *P*. *aeruginosa* PAO1 by Schweizer *et* *al*. (1997). The authors also described a second gene within the same cluster, *glpK*, encoding glycerol kinase – and functionally linking substrate transport with metabolism with the genomic architecture of the cluster. While the GlpT protein of *E*. *coli* is a *sn*‐glycerol‐3‐*P*/inorganic phosphate antiporter (Lemieux *et* *al.,* 2005), the GlpT transporter present in some *Pseudomonas* species (such as *P*. *aeruginosa* PAO1 and *P*. *fluorescens* SBW25) seems to act as a dual *sn*‐glycerol‐3‐*P*/fosfomycin symporter (Hirakawa *et* *al.,* 2018). Such a mechanism has not been identified in *P*. *putida* KT2440.

**Figure 2:**
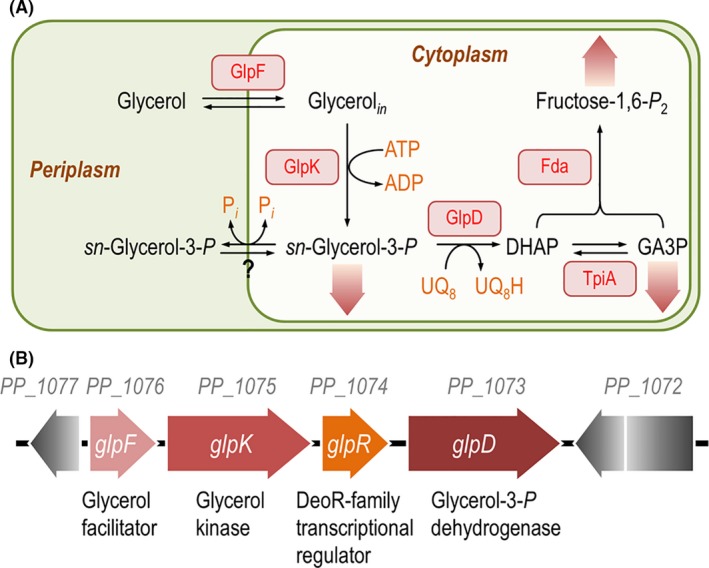
Biochemical pathways and genetic organization of genes involved in glycerol metabolism in *Pseudomonas putida* KT2440. A. Main biochemical reactions relevant for glycerol transport, phosphorylation and oxidation of metabolic intermediates thereof. The incorporation of fructose‐1,6‐*P*_2_ and glyceraldehyde‐3‐*P* (GA3P) into central carbon metabolism *via* gluconeogenesis and downward catabolism, respectively, is indicated by a wide shaded arrow. The question mark (?) denotes a potential *sn*‐glycerol‐3‐*P* transporter, yet to be identified in strain KT2440. DHAP, dihydroxyacetone‐*P*; UQ_8_ and UQ_8_H, oxidized and reduced forms (respectively) of ubiquinone 8; and P_*i*_, inorganic phosphate. B. Genetic organization of the *glp* locus in *P*. *putida* KT2440. The genomic region encompasses *glpF* [*PP_1076*, major intrinsic protein (MIP) family channel protein, aquaglyceroporin], *glpK* (*PP_1075*, glycerol kinase), *glpR* (*PP_1074*, DeoR family transcriptional regulator) and *glpD* (*PP_1073*, aerobic *sn*‐glycerol‐3‐*P* dehydrogenase). The *glp* cluster is flanked upstream by *PP_1072*, which encodes an uncharacterized leucine‐rich repeat‐containing protein, and downstream by *PP_1077*, encoding an YbaK/EbsC‐type protein [prolyl‐tRNA editing protein, probably a Cys‐tRNA(Pro) deacylase] (Nelson *et* *al.,* 2002; Belda *et* *al.,* 2016). The elements in this outline are not drawn to scale.

By gathering genetic information, the pathway for glycerol metabolism was reconstructed for both *P*. *aeruginosa* and *P*. *putida*, and it was found to be similar to the set of aerobic biochemical reactions for glycerol processing in *E*. *coli* (shown in Fig. 2A for *P*. *putida* KT2440). The sequence of reactions catalysed by the ATP‐dependent GlpK kinase and the ubiquinol‐dependent GlpD dehydrogenase generates DHAP, serving both as the point of entry of glycerol into central carbon metabolism and the driving force for substrate transport and consumption. DHAP, in turn, is split essentially into gluconeogenesis (*via* fructose‐1,6‐*P*_2_) and downward catabolism (*via* glyceraldehyde‐3‐*P*, GA3P; see also Fig. 2A). No enzymes similar to either GlpABC or GldA of *E*. *coli* (see Fig. 1) have been identified thus far in *Pseudomonas* species, indicating that oxygen‐dependent pathways for glycerol utilization is the preferred route in this genus [characterized by the abundance of strictly‐aerobic species (Silby *et* *al.,* 2011; Nikel *et* *al.,* 2014a, 2014b)].

The relatively simple biochemistry underlying glycerol utilization is reflected in a rather conserved genetic architecture of the *glp* genes across species, with *P*. *putida* KT2440 as an archetypal example (Fig. 2B). In strain KT2440, the genes deemed essential for glycerol metabolism are arranged in a genomic cluster that includes *glpF* (*PP_1076*, aquaglyceroporin), *glpK* (*PP_1075*, glycerol kinase), *glpR* (*PP_1074*, a transcriptional regulator belonging to the DeoR family) and *glpD* (PP_1073, the main *sn*‐glycerol‐3‐*P* dehydrogenase). Other genes with plausible roles in glycerol processing and metabolism and annotated as such in the *Pseudomonas* database are *gpsA* [*PP_4169*, encoding a soluble, NAD(P)^+^‐dependent *sn*‐glycerol‐3‐*P* dehydrogenase, poorly studied in microorganisms but likely involved in glycerophospholipid breakdown as a glyceroneogenic enzyme (Reshef *et* *al.,* 2003)] and several glycerol/*sn*‐glycerol‐3‐*P* acyl transferases (Winsor *et* *al.,* 2016). The structural organization of the *glp* gene cluster is highly conserved in both *P*. *putida* KT2440 and *P*. *aeruginosa* PAO1 – although the relative orientation of the genes is inverted. Furthermore, the *glp* gene cluster of strain KT2440 (i.e. *PP_1076* to *PP_1073*) exhibits a high degree of sequence identity with *glpF* (83%), *glpK* (82%), *glpR* (80%) and *glpD* (72%) of *P*. *aeruginosa* PAO1 (*PA_3581* to *PA_3584*). As indicated in the next sections, there is an intimate relationship between the genetic organization of the *glp* genes, the transcriptional regulation exerted by the GlpR protein, and the biochemical network deployed by *Pseudomonas* when cells are growing on glycerol.

### Growth on glycerol promotes a mixed gluconeogenic and glycolytic regime *in the metabolism* of Pseudomonas

**Figure 3:**
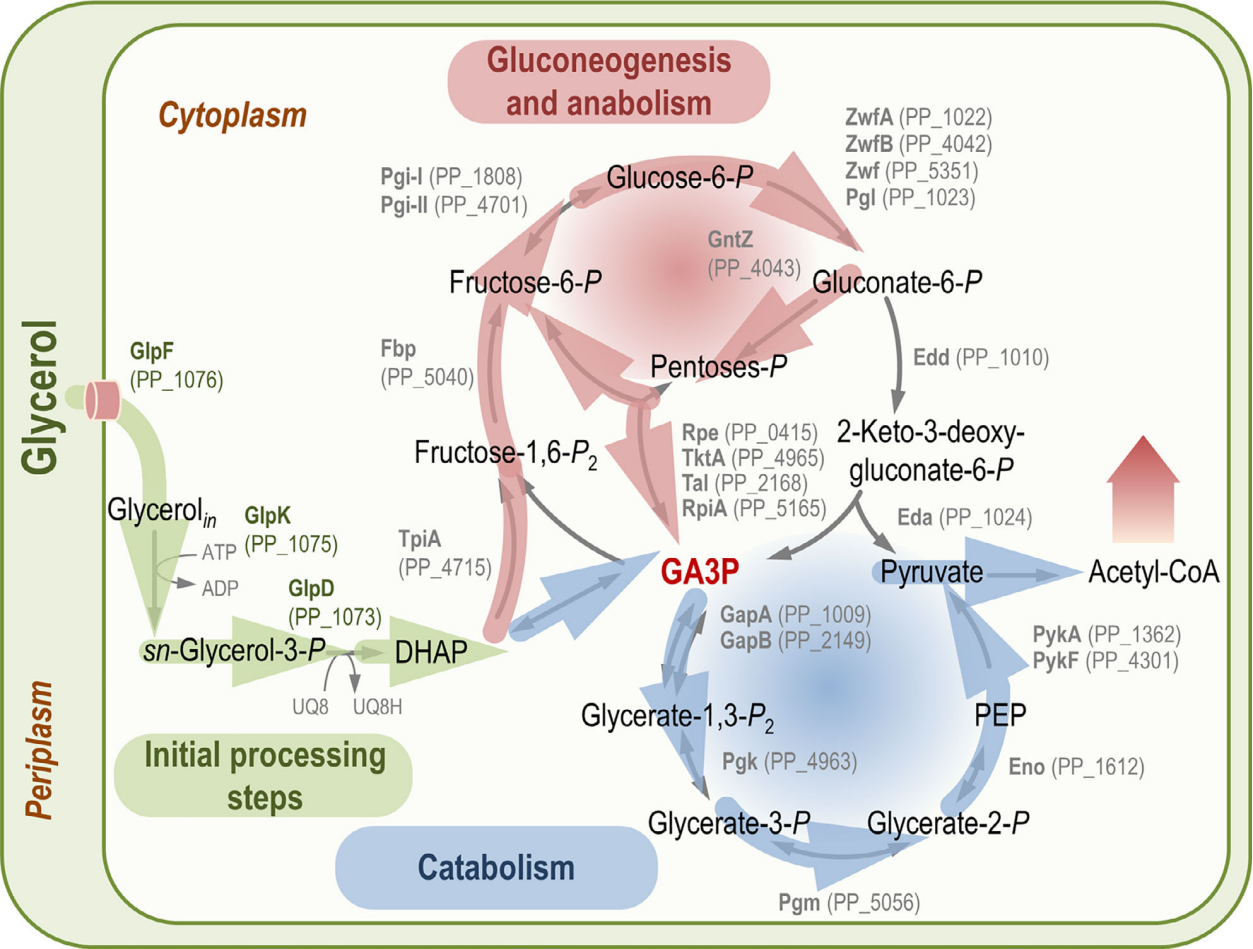
The metabolism of glycerol in *Pseudomonas putida* KT2440 involves a combination of special processing pathways coupled to both glycolytic and gluconeogenic routes. Reactions within the upstream central carbon metabolism in strain KT2440 affected by growth on glycerol as indicated by transcriptome and metabolic flux analyses are shown in this scheme. The biochemical network sketches the main pathways involved in carbon processing along with the enzymes catalysing the corresponding conversions. In some cases, reactions have been lumped to simplify the diagram (e.g. within the non‐oxidative pentose phosphate pathway), and only some isoforms of the corresponding enzymes are shown. Further metabolism of acetyl‐coenzyme A (acetyl‐CoA) is indicated by a wide shaded arrow. Glyceraldehyde‐3‐*P* (GA3P) is highlighted as a key node connecting the main metabolic blocks (indicated in the diagram as ‘initial processing steps’, ‘gluconeogenesis and anabolism’ and ‘catabolism’) active in glycerol‐grown cells. DHAP, dihydroxyacetone‐*P*; PEP, phosphoenolpyruvate. The transcriptomic and fluxomic data used in this diagram have been gathered from Nikel *et* *al*. (2014a), Nikel *et* *al*. (2015b) and Beckers *et* *al*. (2016).

With the onset of considering glycerol as a relevant substrate for biotechnological processes, several studies have examined how *Pseudomonas* species react to this compound at different levels. Nikel *et* *al*. (2014a) analysed the similarities and divergences in the use of glycerol by *P*. *putida* with respect to other bacteria by adopting a transcriptomic approach based on deep sequencing of mRNA transcripts complemented by traditional biochemical assays. The main conclusion of that study is that growth on glycerol imposes a particular metabolic response in *P*. *putida* characterized by the activation of both glycolytic and gluconeogenic routes (Fig. 3). The most salient features of the genome‐wide response to the substrate include (i) the transcriptional upregulation of glycerol transport and catabolic genes (i.e. the *glp* gene cluster), (ii) the downregulation of alternative routes for carbon processing, (iii) the activation of a general gluconeogenic response and (iv) the concomitant slow‐down of activities through the tricarboxylic acid (TCA) cycle and the gluconate/2‐ketogluconate loop for oxidative processing of hexoses. The glycerol‐consuming status seems therefore to favour biomass build‐up while preventing loss of carbon as CO_2_ or during the formation of oxidized by‐products [e.g. some organic acids typically produced when *Pseudomonas* cells are grown on sugars (Fuhrer *et* *al.,* 2005)]. Apart from these general physiological features, several regulatory nodes can be identified in the biochemical network that enable efficient and tightly‐controlled substrate utilization.

The Mg^2+^‐ and ATP‐dependent phosphorylation of glycerol to *sn*‐glycerol‐3‐*P* catalysed by GlpK is the key regulatory and rate‐limiting step in glycerol utilization in *E*. *coli* (Zwaig *et* *al.,* 1970). In this species, GlpK activity is modulated by multiple factors, e.g. ATP concentration, allosteric inhibition mediated by fructose‐1,6‐*P*_2_, and direct inhibition by the IIA^Glc^ cytosolic component of the sugar phosphotransferase system (Applebee *et* *al.,* 2011). It is plausible that some of these regulatory features are kept in *Pseudomonas* species – with the likely exception of the interplay with the sugar phosphotransferase system, since glucose transport in *Pseudomonas* proceeds through a different mechanism (del Castillo *et* *al.,* 2007; Daddaoua *et* *al.,* 2009; Pflüger‐Grau and de Lorenzo, 2014). In addition to the enzymatic regulation of the components of glycerol catabolism themselves, more general regulatory patterns are at play in central carbon metabolism.

As indicated in the previous section, DHAP is a key metabolite connecting glycerol with the core metabolism. Downstream catabolism proceeds through the processing of GA3P *via* the activity of GA3P dehydrogenase. The genome of *P*. *putida* KT2440 encodes two *bona fide* GA3P dehydrogenase isozymes, i.e. GapA (PP_1009) and GapB (PP_2149), which are easily identified given their similarity to the same enzyme counterparts in related microorganisms. Because of the reversibility of the oxidation step of GA3P into glycerate‐1,3‐*P*_2_, GA3P dehydrogenase plays a pivotal role acting either on its downward mode [i.e. glycolysis, funnelling GA3P into the Embden‐Meyerhof‐Parnas (EMP) pathway] and in gluconeogenesis (Lessie and Phibbs, 1984). This biochemical step lies at the very core of both glycolytic and gluconeogenic metabolic pathways in most microorganisms, deciding the direction in which the carbon flow proceeds. Apart from GapA and GapB, strain KT2440 possesses two other GA3P dehydrogenase isozymes (encoded by *PP_0665* and *PP_3443*). RNA sequencing indicated that *gapB*,* PP_0665* and *PP_3443* are transcriptionally affected by the presence of glycerol. While PP_0665 does not seem to contribute to the total GA3P dehydrogenase activity in glycerol‐grown *P*. *putida*,* in vitro* biochemical analyses with a Δ*PP_3443* derivative of strain KT2440 accredits a role for PP_3443 as the source of a GA3P dehydrogenase activity relevant for glycerol metabolism, and its cofactor dependence (NADP^+^) points to a likely gluconeogenic role (Nikel *et* *al.,* 2014a).

Glycerol metabolism relies on functional and active sugar catabolic pathways in *P*. *aeruginosa* PAO1 (Blevins *et* *al.,* 1975; Heath and Gaudy, 1978). One of the key nodes for metabolic regulation of glycerol utilization is the activity of GA3P dehydrogenase, which appears to require an active hexoses‐*P* metabolism. Accordingly, genes encoding enzymes within the gluconeogenic branch of the EDEMP cycle and the pentose phosphate (PP) pathway in *P*. *putida* KT2440 were found to be transcriptionally stimulated by growth on glycerol (Fig. 3). Furthermore, since HexR (PP_1021) is a transcriptional repressor controlling genes encoding key steps of these routes, including *gapA* (Udaondo *et* *al.,* 2018), there is a close connection between the use of sugars and glycerol as carbon sources. The metabolite 2‐keto‐3‐deoxy‐6‐phosphogluconate, an intermediate of the Entner‐Doudoroff (ED) pathway (Nikel *et* *al.,* 2015a), acts as an specific effector of the HexR protein (del Castillo *et* *al.,* 2008) – which further supports the role of an active EDEMP cycle in enabling glycerol utilization.

### The characteristic growth phenotype of *Pseudomonas putida* in glycerol cultures

When *P*. *putida* KT2440 is grown in a minimal medium containing glycerol, the specific growth rate attained by the cultures is ca. 30% lower than that of glucose‐grown cultures; conversely, the yield of biomass on substrate increases by ca. 24% (Nikel *et* *al.,* 2014a). Hintermayer and Weuster‐Botz (2017) simulated growth parameters of strain KT2440 *in silico* considering 57 individual carbon sources, and experimentally validated their prediction on six of them (acetate, glycerol, citrate, succinate, malate and CH_3_OH). Glycerol was found to promote the highest biomass yield on substrate (0.61 C‐mol C‐mol^−1^). This feature indicates that this substrate can promote high yields not only of cell components, but also metabolites and products derived from actively growing cells (i.e. primary metabolites).

A phenomenon consistently observed in glycerol cultures is a considerable lag phase (Escapa *et* *al.,* 2012a; Nikel *et* *al.,* 2014a), which has been interpreted as the macroscopic consequence of a substantial re‐arrangement of the whole metabolic network prior to reaching an optimum for growth on this substrate. Closer examination of the phenomenon revealed a stochastic transcriptional response of the *glp* genes as explained later in this article. In any case, RNA sequencing in cells harvested from these cultures indicated a general decrease in the transcription of genes encoding stress response components, further accompanied by the differential expression of elements of the respiratory chain. This raises interesting questions on the relationship between growth rate, stress and the general fitness in Gram‐negative bacteria. It has been suggested that microorganisms are subjected to the general biological principle of *caloric restriction*, i.e. highly energetic carbon substrates lead to transient fast growth – but also to physiological stress and a relative loss of individual reproductive capacity (Skinner and Lin, 2010). The overall physiology of glycerol‐grown *P*. *putida* is consistent with such a perspective: by avoiding to overrun the reactions within the TCA cycle and peripheral (oxidative) metabolic loops, and by recycling carbon equivalents to biomass building blocks, cells may grow slower in glycerol and be less energized. Yet, under these circumstances, the impact of metabolism would not be highly stressful – and the population as a whole should be eventually more successful in terms of final numbers. The fact that glycerol itself acts as an osmoprotectant (Sleator and Hill, 2002) indicates that growth on the polyol determines a less stressful cell physiology as compared to the use of sugars as a carbon source. Accordingly, the maintenance coefficient of *P*. *putida* KT2440 growing on glycerol has been determined to be 0.039 mmol_substrate_ g_cell dry weight_^−1^ h^−1^ (Beckers *et* *al.,* 2016), ca. 35% lower than that observed in cells grown on glucose (Ebert *et* *al.,* 2011). In all, such physiological situation is reflected in a decreased swimming motility, a coarse descriptor of the energy load of the cells, when *P*. *putida* KT2440 is grown on glycerol as compared to sugars or TCA cycle intermediates (Nikel *et* *al.,* 2014a). Scoffield and Silo‐Suh (2016) recently reported that glycerol metabolism promotes biofilm formation by both a chronic cystic fibrosis isolate and a wound isolate of *P*. *aeruginosa*, linking caloric restriction to pathogenesis (La Rosa *et* *al.,* 2018). Moreover, loss of the GlpR regulator, enhanced biofilm formation through the upregulation of genes encoding enzymes needed to synthesize the Pel polysaccharide – with a concomitant decrease of energy‐expensive motility. Similarly, when *P*. *fluorescens* was subjected to an oxidative challenge with hydrogen peroxide in a mineral medium containing glycerol as the sole carbon source, the bacterium reconfigured its metabolism to generate ATP primarily *via* substrate level phosphorylation, with the concomitant synthesis of large amounts of phosphoenolpyruvate and pyruvate (Alhasawi *et* *al.,* 2016). The overall phenomenon of metabolic reconfiguration, which deserves further investigation across different bacterial species, seems to constitute an evolutionary trait that enables *Pseudomonas* species to tune the balance prevalence‐*versus*‐niche exploration depending on the available carbon sources. Both the specific and general physiological and metabolic responses to glycerol have been explored in *P*. *putida* also under different growth schemes, including chemostat cultures, as disclosed in the next section.

### Glycerol utilization analysed from a systems biology perspective

Environmental bacteria have developed remarkable regulatory systems, which allow them to thrive and cope with various environmental conditions such as extreme temperatures, exposure to metals and nutrient availability, to name but a few of them (Domínguez‐Cuevas *et* *al.,* 2006; Daniels *et* *al.,* 2010; Krell *et* *al.,* 2012; Tribelli *et* *al.,* 2013; de Lorenzo *et* *al.,* 2015; Belda *et* *al.,* 2016; Chavarría *et* *al.,* 2016). Bacteria display an exquisitely fine‐tuned modulation of gene expression with the aim to maintain cellular functions, with this regulation occurring both at the transcriptional and the post‐transcriptional level (Arce‐Rodríguez *et* *al.,* 2016). These regulatory programs control hundreds of enzymatic reactions, fuelling metabolism and sustaining bacterial growth on a variety of nutritional situations (Schuetz *et* *al.,* 2012). How nutrient availability drives global gene expression in bacterial species has been an important area of study in the last decade (Chubukov *et* *al.,* 2013; Kohlstedt *et* *al.,* 2014; Vital *et* *al.,* 2015). As indicated above, *Pseudomonas* species have received special attention when grown on glycerol as the sole carbon source under different fermentation modes (Wang and Nomura, 2010; Kim *et* *al.,* 2013; Licciardello *et* *al.,* 2017). It is important to highlight that the physiology of cells growing in batch cultures (i.e. in the presence of excess substrate) highly differs from that in continuous cultivation setups in terms of gene expression (transcriptome), protein abundance (proteome) and conversion rates in biochemical reactions (fluxome) – which ultimately define growth patterns and macroscopic phenotypes. Chemostats are an excellent tool to evaluate physiological parameters since constant growth rates can by externally adjusted by the operator through the dilution rate (*D*) while maintaining other relevant parameters strictly controlled (e.g. pH, oxygen levels and nutrients concentration).

Against this background, Beckers *et* *al*. (2016) recently elucidated gene expression and metabolic flux patterns in *P*. *putida* KT2440 grown on glycerol under different growth regimes and nutrient‐limiting conditions. Genes belonging to the oxidative PP pathway (*zwfA*), ED pathway (*eda*) and the pyruvate node (*acoABC*, encoding the components of a dual dehydrogenase) showed higher expression levels by changing the imposed specific growth rate (from 0.044 to 0.12 h^−1^) under carbon limitation conditions – echoing the results previously observed in batch cultures with glycerol (Nikel *et* *al.,* 2014a). Remarkably, this was not the case for the same genes of the PP and ED pathways when their expression was examined under nitrogen limitation: when shifting from carbon to nitrogen limitation, the mRNA levels of genes of the PP and ED pathways showed no changes, and gene encoding elements of both the pyruvate node and isocitrate dehydrogenase (encoded by *icd*,* PP_4012*) were downregulated (Beckers *et* *al.,* 2016); see also Fig. 3. Analysis of the metabolic flux *via* the PP and ED pathways corroborated the findings from the transcriptome analysis, with a strong dependence on the activity of the EDEMP cycle when cells were grown under nitrogen limitation at *D *=* *0.12 h^−1^.

The scenario described above is somewhat different to the one observed in *P*. *putida* LS46 grown in glycerol‐containing batch cultures (Fu *et* *al.,* 2015). By comparing nitrogen *versus* carbon limitation, the authors have found that the transcription of genes encoding pyruvate dehydrogenase and isocitrate dehydrogenase was upregulated, and proteomic analysis supported this observation at the enzyme abundance level. In addition, carbon fluxes through the glyoxylate shunt (usually inactive in the presence of glucose) were found to be extremely high under nitrogen limitation, giving rise to two industrially important by‐products, succinate and malate. These observations indicate that the pattern of metabolic regulation differs among strains, and furthermore highlight the relevance of glycerol as a substrate for the synthesis of reduced bioproducts. The next relevant question is how the overall cell physiology in glycerol‐grown *P*. *putida* is tied to the unique transcriptional signature imposed by the GlpR regulator – an issue examined in the following section.

### The transcriptional activation of the glp gene cluster follows a bimodal regime and defines the glycerol‐dependent growth pattern of *P. putida*: a unique case of metabolic persistence

The emergence of methodologies designed to study bacteria at the single cell level revealed a complete repertoire of responses of individual microorganisms to specific environmental cues (Ackermann, 2015; Roberfroid *et* *al.,* 2016; Osella *et* *al.,* 2017). These observations challenge the customary view of prokaryotic growth and metabolism as a homogeneous, co‐occurring process in space and time. The phenomenon broadly known as *persistence*, i.e. the occurrence of a live but non‐growing fraction of cells within a bacterial population (van den Bergh *et* *al.,* 2017), is a prime example in this respect. While the lack of microbial growth may appear negative at a first glance, persistence ensures the survival of cells exposed to agents hitting developing bacteria, e.g. antibiotics. Once the selective pressure ceases, persistent bacteria can resume growth and fully restore the original population (Balaban *et* *al.,* 2004). Regardless of the mechanisms behind this phenomenon, the standing question is whether persistence is to be considered as an adaptive trait or a casual occurrence. What we qualify as persistence may just be a particular case of a more common situation in which a starting population stochastically splits between growing and non‐growing cell types when facing a new set of environmental or physicochemical conditions (Cabral *et* *al.,* 2018). Environmental bacteria are also subjected to clonal and phenotypic variability (van den Broek *et* *al.,* 2005; Volke and Nikel, 2018; Schiessl *et* *al.,* 2019), especially when growing on alternative substrates such as aromatic compounds (Nikel *et* *al.,* 2014c) – but our studies on glycerol utilization by *P*. *putida* revealed that simpler carbon substrates can likewise elicit a similar stochastic response.

**Figure 4:**
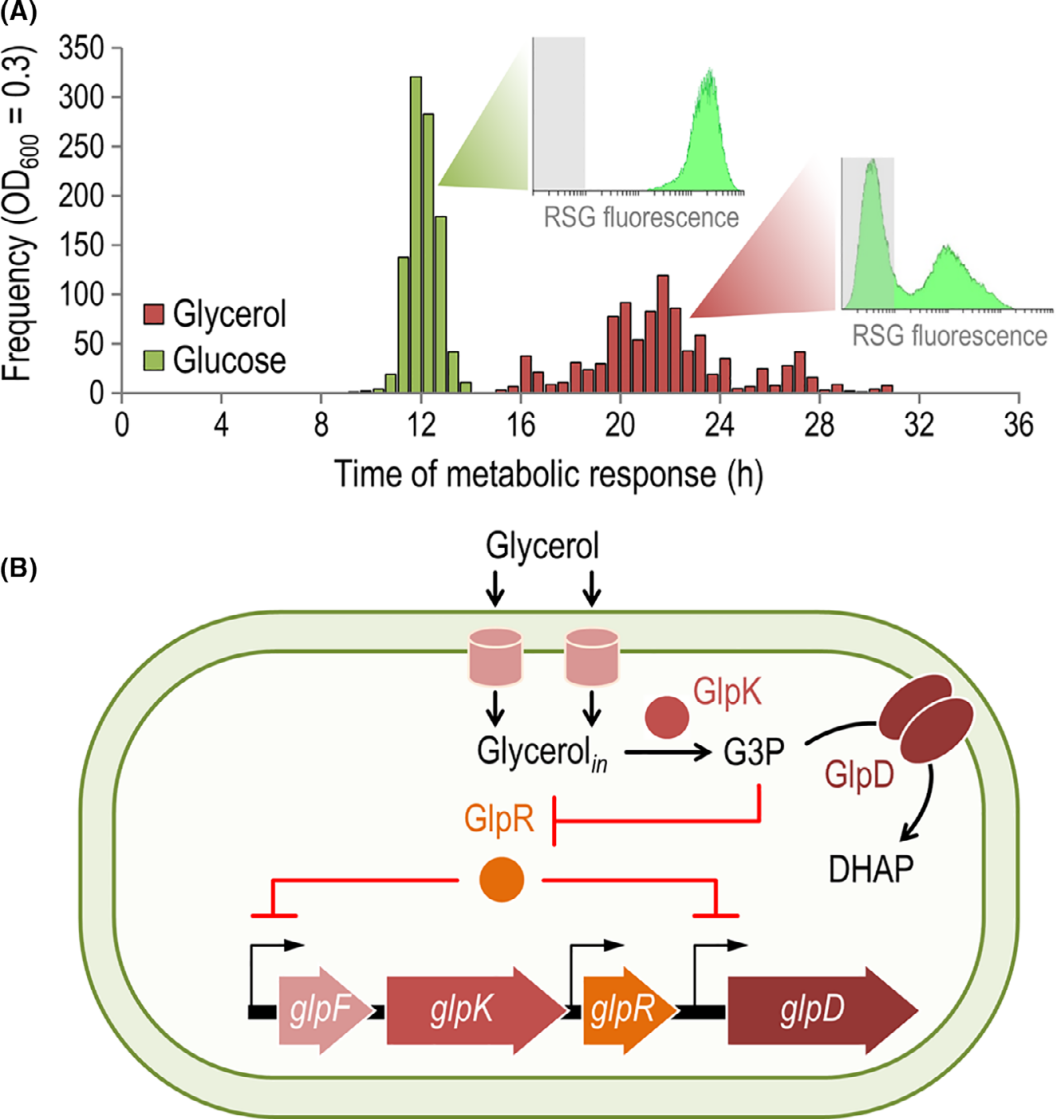
The genetic wiring of genes involved in glycerol utilization in *Pseudomonas putida* KT2440 sets a bimodal metabolic regime in the bacterial population. A. A protracted lag phase is observed when cells are grown on glycerol (which is not detected on glucose). The *time of metabolic response* is defined as the period needed to reach an optical density at 600 nm (OD_600_) = 0.3 (i.e. mid‐exponential growth), and its frequency distribution is shown here for 1,000 independent single‐cell batch cultures run in microtitre plates with either glycerol or glucose as the carbon source. Note the bimodal nature of the distribution of times of metabolic response in glycerol cultures. As indicated in the inset plots, the Bac*Light*™ *RedoxSensor*™ Green (RSG) reagent in combination with flow cytometry enables the identification of different bacterial populations by their level of metabolic activity (Corona *et* *al.,* 2018). The grey rectangle in each histogram plot identifies the region considered negative for the RSG fluorescence signal. Data taken from Nikel *et* *al*. (2015b) and Nikel and de Lorenzo (2018a). B. The lag phase observed in glycerol cultures can be traced to the regulatory architecture of the *glp* gene cluster, as the product of the first reaction [*sn*‐glycerol‐3‐*P* (G3P)] is needed to release the repression exerted by GlpR. As the expression of *glpF* and *glpK* is also repressed by the regulator, the only way to form G3P is through the low‐probability effector‐independent stochastic lifting of the GlpR‐mediated repression. This particular transcriptional wiring thus translates into different levels of metabolic activity. DHAP, dihydroxyacetone‐*P*.

A noteworthy feature consistently detected in glycerol cultures is an anomalously long lag phase (typically lasting > 10 h) before any noticeable growth is evident (Escapa *et* *al.,* 2012a; Nikel *et* *al.,* 2014a) – an occurrence not observed when the cells are cultured on glucose or succinate under the same conditions. Exposure of strain KT2440 to glycerol leads to the appearance of two sub‐populations that differ in their level of metabolic activity towards the carbon substrate, and the relative proportion of these bacterial sub‐populations (i.e. active and inactive) changes over time (Nikel *et* *al.,* 2015b). The phenomenon has been studied by defining the so‐called *time of metabolic response*, which identifies the stretch needed for single‐cell cultures to reach an optical density at 600 nm (OD_600_) of 0.3 units, i.e. corresponding to mid‐exponential growth (Nikel and de Lorenzo, 2018a). By systematically recording OD_600_ values in 1000 independent, single‐cell microtitre‐plate cultures of *P*. *putida* KT2440 grown on either glucose or glycerol, the distribution of times of metabolic response was plotted as a function of the time elapsed since inoculation (Fig. 4A) – clearly identifying the existence of more than one bacterial sub‐population in glycerol cultures. Flow cytometry‐assisted analysis of the overall level of metabolic activity in these cultures supported this notion: glycerol cultures were characterized by the presence of a dormant fraction of bacterial cells coexisting with a metabolically active sub‐population, whereas a single, uniform and metabolically active *P*. *putida* population was observed when cells were grown in the presence of glucose. This phenomenon seems to represent the mirror counterpart of persistence, i.e. the stochastic rise of individual cells able to metabolize the substrate amidst a majority of glycerol‐unresponsive bacteria, followed by the eventual take‐over of the entire *P*. *putida* population.

Elucidation of the functional interactions between glycerol‐derived metabolites and the transcriptional architecture of the *glp* gene cluster in strain KT2440 provided an explanation for the macroscopic phenotype of cells grown on glycerol cultures (Fig. 4B). While virtually all prokaryotic promoters are subject to a degree of noise (Elowitz *et* *al.,* 2002), certain regulatory devices translate such noise into bi/multi‐modal or bi‐stable manifestation of the corresponding phenotypes in single cells. Cells will start growing only if the low‐probability effector‐independent stochastic lifting of the GlpR‐mediated repression allows for the expression of *glpF* and *glpK* (the latter gene encoding the kinase responsible of *sn*‐glycerol‐3‐*P* formation). Once this repression is stochastically defeated, the full expression of the *glp* genes can proceed – finally returning to an *OFF* state when the substrate is completely depleted. Different levels of metabolic activity are observed in the cells, reflecting their ability to catabolize glycerol, while the transcriptional derepression process is undergoing. This situation, in turn, explains the very long lag phase in *P*. *putida* cultures on glycerol. Further confirmation of this hypothesis comes from (i) deletion of *glpR* and (ii) controlled overexpression of *glpFK* – both manipulations resulting in the disappearance of the protracted lag phase on glycerol, and in the uniform distribution of growth phenotypes (Nikel *et* *al.,* 2015b). The prolonged unresponsiveness of cells exposed to glycerol could enable carbon source‐dependent *metabolic bet‐hedging* to explore new chemical and nutritional landscapes; a concept reminiscent of foraging in animal ecology, in which some members of the population (but not the *entire* population) take risks to broaden the search for alternative food sources. Under this scheme, the cost of randomly expressing metabolic genes in *P*. *putida* is outweighed by the potential benefit of locating (and being prepared to utilize) alternative carbon sources such as glycerol, which is not usually present at high concentration in environmental niches colonized by *Pseudomonas*. After discussing the intricate combination of biochemical and genetic mechanisms of regulation in glycerol‐grown *Pseudomonas*, we now move onto another relevant aspect of this compound, i.e. its value as a carbon substrate in biotechnological processes.

## Biotechnology of glycerol valorization by *Pseudomonas* species

**Figure 5:**
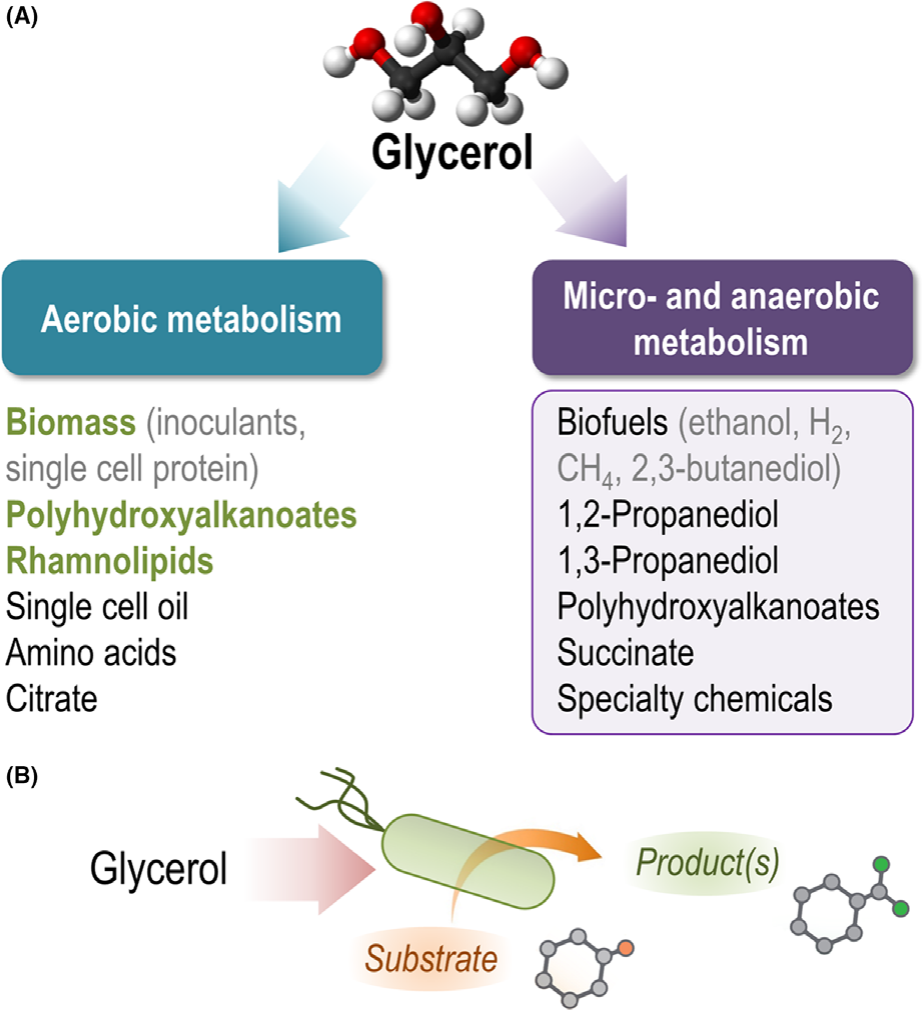
Glycerol as a substrate for biotechnology using *Pseudomonas*. A. Several *Pseudomonas* species have been adapted as the biocatalyst for the bioproduction of a number of products derived from glycerol, using either wild‐type strains or engineered variants thereof. Several products that could be obtained from this substrate are listed, and those already synthesized from glycerol in *Pseudomonas* species are highlighted in green (see also Table 1 and its description in the text for further details). Note the (relatively untapped) potential of micro‐ and anaerobic metabolism for bioproduction of value‐added compounds from glycerol – some of which are likewise produced in aerobic bioprocesses. B. Apart from its prominent role as a carbon source, glycerol can also be used to fuel energy metabolism in resting cells to mediate specific biotransformations. In this case, glycerol‐grown, wild‐type or engineered *Pseudomonas* cells execute a biotransformation (e.g. a stereoselective reduction) on an externally added substrate.

Microbial fermentations using glycerol as the main carbon source have been exploited for the production of a wide variety of value‐added compounds, ranging from simple molecules to structurally complex polymers (Fig. 5A). In some practical cases, glycerol has been used to promote *Pseudomonas*‐based biotransformation processes (Fig. 5B), in which the biocatalyst executes a given biochemical reaction fuelled by the addition of a carbon source besides the substrate being transformed. While all the examples available in the literature describe aerobic processes for glycerol valorization, the possibility of engineering a micro‐ or anaerobic metabolism in *P*. *putida* remains a fascinating – and challenging (Nikel and de Lorenzo, 2013) – possibility that could open new avenues for biotechnological production (Fig. 5B). In the sections below and Table 1, we present some of the most relevant examples on the use of glycerol as the main substrate for the production of value‐added molecules by *Pseudomonas* species.

**Table 1:** Selected examples of bioprocesses for the production of value‐added biochemicals using *Pseudomonas* strains and glycerol as the main carbon substrate

Strain	Product	Titre (g l^−1^)	Yield (g g^−1^)	Productivity (g_product_ l^−1^ h^−1^)	Fermentation mode	References
*P*. *putida* GO16	mcl‐PHA^a^	6.3	0.16	0.13	Fed‐batch	Kenny *et* *al*. (2012)
*P*. *putida* KT2440	mcl‐PHA	1.5	0.05	0.02	Batch	Poblete‐Castro *et* *al*. (2014)
*P*. *putida* KT2440 Δ*phaZ*	mcl‐PHA	2.0	0.07	0.03	Batch	Poblete‐Castro *et* *al*. (2014)
*P*. *putida* LS46	mcl‐PHA	0.6	0.02	0.01	Batch	Fu *et* *al*. (2014)
*P. mosselii* TO7	mcl‐PHA	1.3	N.A.	0.03	Batch	Liu *et* *al*. (2018)
*P. aeruginosa*	Rhamnolipids	8.9	0.08	0.04	Batch	Sodagari *et* *al*. (2018)
Engineered *P. putida* KT2440	Rhamnolipids	1.1	N.A.	0.05	Batch	Tiso *et* *al*. (2017)
Engineered *P. putida* KT2440	2‐Oxocarboxylates^b^	8.2	N.A.	1.4	Batch	Wang *et* *al*. (2015)
Engineered *P. putida* S12	*p*‐Hydroxybenzoate	1.8	0.39	0.03	Fed‐batch	Verhoef *et* *al*. (2007)
Engineered *P. taiwanensis*	Phenol	0.4	0.09	0.005	Batch	Wynands *et* *al*. (2018)
Engineered *P. chlororaphis*	*cis,cis*‐muconate	3.4	0.19	0.03	Fed‐batch	Wang *et* *al*. (2018)
Engineered *P. chlororaphis*	Phenazine‐1‐carboxamide	9.2	N.A.	0.19	Batch	Peng *et* *al*. (2018)
Engineered *P. chlororaphis*	Phenazine‐1‐carboxamide	4.1	0.23	0.12	Batch	Yao *et* *al*. (2018)
Engineered *P. denitrificans*	3‐Hydroxypropionate	5.0	0.67	0.12	Batch	Zhou *et* *al*. (2013)
Engineered *P. putida* S12	Butanol	0.2	0.04	0.01	Batch	Nielsen *et* *al*. (2009)
Engineered *P. putida* KT2440	*N*‐Methylglutamate	17.9	0.11	0.13	Fed‐batch	Mindt *et* *al*. (2018)
*P. fluorescens* Δ*mucA*	Alginate	7.9	N.A.	0.11	Batch	Maleki *et* *al*. (2017)
*P. putida* PCL1445	Lipopeptide	N.A.	N.A.	N.A.	Batch	Dubern and Bloemberg (2006)
	Putisolvin					
*P. fluorescens* BD5	Pseudofactin	1.2	N.A.	0.01	Batch	Biniarz *et* *al*. (2018)

N.A., not available.

**a**. In this context, mcl‐PHA indicates any type of medium‐chain‐length polyhydroxyalkanoate, although the exact composition of the polymers differs in different studies according to culture conditions.

**b**. Biotransformation.

### Polyhydroxyalkanoates – industrial biopolymers

There is little doubt that one of the biggest challenges that modern society is facing is the use of non‐renewable materials for the production of fine and bulk chemicals at the industrial scale (Becker and Wittmann, 2018; de Lorenzo *et* *al.,* 2018; Dupont‐Inglis and Borg, 2018). When glycerol emerged as a promising feedstock for bacterial fermentation in the last decade, the bioconversion of this substrate into polyhydroxyalkanoates (PHAs), a family of biopolymers with similar mechanical and physical properties to that of synthetic thermoplastics, became an immediate goal. Many bacterial species synthesize PHAs as carbon and energy storage compounds under growth conditions characterized by an abundance of carbon sources with respect to other nutrients, such as nitrogen or phosphorus (Anderson and Dawes, 1990; Gomez *et* *al.,* 2012; López *et* *al.,* 2015). The physicochemical properties of these polymers (e.g. thermoplastic properties, and hence, industrial applicability) largely depend on the size (i.e. chain length) of the monomer (Meng and Chen, 2018). The most common and widespread PHA is poly(3‐hydroxybutyrate), but several bacteria are known to accumulate PHAs with monomers of lengths between 3 and 20 carbon atoms when fed with specific substrates (Leong *et* *al.,* 2014). Polymers composed by C3‐C5 monomers are called *short‐chain‐length PHAs* (scl‐PHAs), whereas *medium‐chain‐length PHAs* (mcl‐PHAs) contain C6‐C14 monomers. Long‐chain‐length PHAs have monomers longer than C14. These polymers continue to attract increasing industrial interest as renewable, biodegradable, biocompatible, and extremely versatile thermoplastic and elastomeric materials (Suriyamongkol *et* *al.,* 2007; Koller *et* *al.,* 2017). The biochemistry and molecular biology of PHA synthesis and degradation in several bacterial species has been elucidated (Kessler and Witholt, 2001). PHAs are deposited intracellularly as complex inclusion bodies or granules (Grage *et* *al.,* 2009). Polymer granules include, among other proteins, PHA synthase, depolymerizing enzymes, regulatory proteins, and structural proteins termed *phasins* (Mezzina and Pettinari, 2016).

*Pseudomonas* species are natural producers of mcl‐PHAs (Prieto *et* *al.,* 2016; Poblete‐Castro *et* *al.,* 2017), and these polyesters can be accumulated under nutrient imbalance conditions using a broad array of carbon sources, e.g. fatty acids, sugars, waste materials and glycerol (Poblete‐Castro *et* *al.,* 2014). When *Pseudomonas* is used as a cell factory for PHA production, the monomer composition can be tuned depending on the carbon source and the fermentation mode chosen for biopolymer synthesis (Meng *et* *al.,* 2014; Chen and Jiang, 2017; Meng and Chen, 2018), since the class II PhaC polymerase enzyme of *Pseudomonas* accepts a broad range of substrates (Prieto *et* *al.,* 2016). Once glycerol is converted into acetyl‐coenzyme A (CoA) in several steps (Fig. 3), this intermediate is redirected to the *de novo* synthesis of fatty acids, resulting in various precursors for the PHA biosynthesis route (Beckers *et* *al.,* 2016). One of the key enzymes of this process is PhaG (a transacylase), which converts (*R*)‐3‐hydroxyacyl‐acyl carrier protein (ACP) thioesters into (*R*)‐3‐hydroxyacyl‐CoA, the substrate of PHA polymerase – thus linking the *de novo* synthesis of fatty acids with the PHA cycle (Rehm *et* *al.,* 1998; Escapa *et* *al.,* 2012b). The biopolymer obtained thereby consists of a mixture of various monomers, but it was found to be particularly enriched in the 3‐hydroxydecanoate (C10) fraction.

*Pseudomonas* strains can accumulate > 30% of its cell dry weight as PHA when grown on glycerol (Escapa *et* *al.,* 2012a), yet PHA productivities and yields are rather low as compared to those observed when fatty acids are used as substrates (Fu *et* *al.,* 2014). Most studies of mcl‐PHAs synthesis from glycerol have focused on the use of raw glycerol (i.e. the by‐product of the biodiesel industry) as the carbon substrate. Given its high capacity to cope with toxic compounds, e.g. CH_3_OH, present in raw glycerol at relatively high concentrations, *Pseudomonas* cells exhibit essentially the same growth pattern as compared to cultures containing pure glycerol. Fed‐batch culture production of mcl‐PHA in *P. putida* GO16 on raw glycerol resulted in a PHA titre of 6.8 g l^−1^ after 48 h of cultivation (Kenny *et* *al.,* 2012). Various *P. putida* strains have been evaluated for their capacity of producing mcl‐PHA on raw glycerol, and *P. putida* KT2440 was found to be the best performer. Interestingly, citrate was observed to accumulate as a by‐product in the culture broth during the fermentation period, reaching a titre of > 20 g l^−1^ in bioreactor cultivations (Poblete‐Castro *et* *al.,* 2014). By‐product formation is certainly an undesired feature for the efficient production of biopolymers, but citrate seems to be regularly present in glycerol fermentations due to the high amount of carbon used during the process. In an attempt to increase the amount of carbon available for PHA synthesis, metabolic engineering strategies have been applied in *P*. *putida* with the aim of reducing by‐product formation, e.g. by model‐driven engineering of strain KT2440, which yielded as much as twice mcl‐PHA content as compared to the parental strain (Sohn *et* *al.,* 2010; Poblete‐Castro *et* *al.,* 2012b). In a separate study, knocking out *glpR* was shown to result in a ca. twofold increment in the mcl‐PHA content produced by strain KT2440 in shaken‐flask cultivations with glycerol as the carbon source (Escapa *et* *al.,* 2012a). Moreover, deletion of *phaZ*, encoding a PHA depolymerase, resulted in ca. 1.4‐fold higher levels of mcl‐PHA content than the wild‐type strain when using raw glycerol as the only carbon substrate (Poblete‐Castro *et* *al.,* 2014). Based on elementary mode analysis, several genetic targets have recently been proposed attempting to enhance PHA accumulation in *Pseudomonas putida* in the presence of glycerol (Beckers *et* *al.,* 2016). A novel programmable genetic circuit for cell autolysis was developed and tested in glycerol‐grown in *P. putida* KT2440 cells when accumulating mcl‐PHAs at high levels (Borrero de Acuña *et* *al.,* 2017). This efficient cell lytic system was based on the heterologous expression of a peptidoglycan‐disrupting enzyme lysozyme, which was further translocated to the periplasm using a signal peptide of *P*. *stutzeri*. Upon induction under nitrogen‐limiting conditions, > 95% of the cell population showed membrane disruption and ca. 75% of the PHA could be recovered at the end of the fermentation period. The application of synthetic biology and systems metabolic engineering approaches in *Pseudomonas* strains, coupled to in‐depth analysis of the phenotypic outcome of these manipulations, is expected to further boost biopolymer production from glycerol towards economic feasibility.

### Rhamnolipids and other biosurfactants

Some *Pseudomonas* strains can thrive on water‐immiscible substrates, such as alkanes and lipids, by secreting specific amphiphilic compounds called biosurfactants, which reduce the tension and interfacial surface between the immiscible substance and water (di Martino *et* *al.,* 2014; Patel *et* *al.,* 2019). The best‐studied biosurfactants are *rhamnolipids*, i.e. a glycolipid in which one or two molecules of rhamnose are linked to one or two β‐hydroxydecanoate moieties (Desai and Banat, 1997; Wittgens and Rosenau, 2018). *Pseudomonas aeruginosa* and *P. fluorescens* have both been reported to produce rhamnolipids at high titres (up to 100 g l^−1^) using various carbon substrates (Schmidberger *et* *al.,* 2013). Glycerol has been proposed as an efficient feedstock for rhamnolipid production in *P. aeruginosa* (Sodagari *et* *al.,* 2018; Zhao *et* *al.,* 2018). Despite these advances, there is still a major drawback in employing *P. aeruginosa* as a rhamnolipid‐producing platform because of its human‐pathogen nature. To circumvent this problem, a metabolically engineered *P. putida* strain, carrying the rhamnolipid biosynthesis pathway from *P. aeruginosa*, has been developed and achieved a rhamnolipid yield of 0.15 g g^−1^ on glucose (Wittgens *et* *al.,* 2011). The same study also indicated that the maximum theoretical rhamnolipid yield that could be achieved when cells are grown on glycerol is similar to the one attained using glucose, which highlights the use of this carbon source for biosurfactant production.

Bacteria of the genus *Pseudomonas* also produce several lipopeptide biosurfactants that display antimicrobial and emulsifying properties. This structurally complex group of lipopeptides is composed of viscosin, amphisin, surfactin, putisolvin and massetolide A, to name but a few of them, and they vary in the number of amino acid residues present in the chemical structure (de Bruijn and Raaijmakers, 2009). *Pseudomonas fluorescens* and *P. putida* strains have been described as producers of lipopeptides, and their biosynthesis is governed by multi‐modular, non‐ribosomal peptide synthetases, enzymes that catalyse synthesis of important peptide products from a variety of standard and non‐proteinogenic amino acid substrates (de Bruijn *et* *al.,* 2008). *P. putida* PCL1445, for instance, has been shown to synthesize high levels of putisolvin on various carbon sources at low temperature (11°C), and glycerol promoted the highest titres among all substrates tested (Dubern and Bloemberg, 2006).

### Production of aromatic compounds

Like many other value‐added products, aromatic compounds are mainly produced from fossil and other non‐renewable resources using processes that involve toxic precursors (e.g. benzene or toluene), high temperatures and complex reaction sequences (Gosset, 2009; Lee and Wendisch, 2017). *Pseudomonas* species have been proposed as biocatalysts for the production of aromatic compounds (Kuepper *et* *al.,* 2015; Molina‐Santiago *et* *al.,* 2016), partially in view of the fact that members of this bacterial genus are known to be outstanding degraders of the same chemical species (Dvořák *et* *al.,* 2017). Several examples from the literature indicate that *P*. *putida* has been engineered for the production of aromatic compounds that are often extremely toxic to be handled by other microbial hosts, e.g. cinnamate, *p*‐coumarate, *p*‐hydroxybenzoate and phenol (Calero *et* *al.,* 2018). One of the pioneering works in engineering *P. putida* for the biosynthesis of aromatic chemicals was the construction of heterologous pathways leading to l‐tyrosine and *p*‐hydroxybenzoate using the solvent‐tolerant *P. putida* S12 as the biocatalyst (Verhoef *et* *al.,* 2007). When cells were grown on glycerol, *p*‐hydroxybenzoate titres of 0.24 and 1.78 g l^−1^ were attained in batch and fed‐batch processes, respectively. More recently, *P. taiwanensis* VLB120 was engineered by knocking in and out genes of the l‐tyrosine pathway and other routes of aromatic degradation (Jiménez *et* *al.,* 2002), and a phenol‐producing strain was obtained *via* the heterologous expression of an efficient tyrosine phenol‐lyase in a plasmid‐free strain that bears 22 genetic modifications in total (Wynands *et* *al.,* 2018). Inactivation of *pykA* (encoding pyruvate kinase) in the engineered *P. taiwanensis* strain further increased the yield of phenol on glycerol up to an unprecedented 18.5% C‐mol C‐mol^−1^. The potential of *Pseudomonas* species as platforms for aromatic compound production from glycerol is expected to be further explored in the future, as the wealth of catabolic pathways for these chemical structures in the *Pseudomonas* genus offers unique opportunities for engineering novel biosynthesis routes.

### Biosynthesis of other value‐added chemicals

The production of *cis*,*cis*‐muconic acid [(2*E*,4*E*)‐2,4‐hexanedioic acid] has been the subject of intense research, as this dicarboxylic acid is a relevant platform chemical and precursor to terephthalic acid, 3‐ and 2‐hexenedioic acid, 1,6‐hexanediol, ε‐caprolactam and ε‐caprolactone – all of which are building blocks of commercial plastics, resins and polymers (e.g. Nylon‐6,6 *via* adipic acid). The synthesis of *cis*,*cis*‐muconic acid from glycerol has been explored using *P. chlororaphis*, a plant growth promoting species, as the platform strain (Wang *et* *al.,* 2018). The engineering strategy consisted of blocking *cis*,*cis*‐muconic acid conversion into end products that could be further metabolized, and augmenting the supply of metabolic precursors by overexpressing *catA*, the gene encoding the rate‐limiting step of the pathway (catechol 1,2‐dioxygenase). The resulting plasmid‐free *P. chlororaphis* strain was able to synthesize *cis*,*cis*‐muconic acid to a maximal titre of 3.4 g l^−1^ in a fed‐batch process, accompanied with a product yield on glycerol of 0.19 g g^−1^. Although this performance is far below the *cis*,*cis*‐muconic acid titre of 65 g l^−1^ achieved in metabolically engineered *P. putida* from aromatics and a co‐feed of glucose (Kohlstedt *et* *al.,* 2018), synthesis from glycerol remains an interesting possibility for the future if further improvements in product yield and titre are achieved.

Metabolic engineering strategies have also been applied in *P*. *putida* for the glycerol‐dependent production of biofuels. For instance, an engineered variant of the solvent‐tolerant *P. putida* S12 strain capable of producing butanol from glycerol was developed by Nielsen *et* *al*. (2009). This strain synthesized 220 mg l^−1^ of butanol in batch cultures. Although still far from the high titre reached by natural butanol‐producing strains, e.g. from the genus *Clostridium,* the potential of these engineered *Pseudomonas* strains is very high due to their production performance in bioreactors and high tolerance to the toxicity exerted by biofuels to the cells (Rühl *et* *al.,* 2009; Vallon *et* *al.,* 2015; Cuenca *et* *al.,* 2016).

Another industrially relevant chemical recently obtained from engineered *P. fluorescens* is alginate, which is widely used in both the food and pharmaceutical industry. This agent is added to food products as an emulsifier, stabilizer and texture‐improver (Bonnichsen *et* *al.,* 2015). In the pharmaceutical manufacturing sector, alginate is compounded into tablets to speed up their disintegration with the aim to release the medically active components in a more controllable fashion as well as to help protecting the stomach mucosa. The non‐pathogenic *P*. *fluorescens* SBW25 Δ*mucA* has been reported to produce alginate from a wide variety of carbon sources such as glucose, fructose and glycerol. Disruption of this anti‐σ factor regulator (encoded by *PFLU_1468*) enables the transcription of *algU*, essential for alginate biosynthesis. This co‐polymer of (1→4)‐linked β‐d‐mannuronate and its C5‐epimer α‐l‐guluronate has been obtained at high titres in both batch and chemostat cultures of *P*. *fluorescens* SBW25 Δ*mucA*, reaching up to 8 g l^−1^ of polysaccharide (Maleki *et* *al.,* 2017).

3‐Hydroxypropionate is another important platform chemical that has received increasing attention in the last few years, given its use for the production of acrylic acid and acrylamides. Production of this compound from glycerol was obtained by overexpressing a glycerol dehydratase of *K. pneumoniae* into *P. denitrificans*, a natural producer of vitamin B_12_ (Lago and Demain, 1969) – which is needed for the biosynthesis of 3‐hydroxypropionate from glycerol (Zhou *et* *al.,* 2013). Yet, there is still a major drawback in using *P. denitrificans* as a host for engineering biosynthetic pathways for 3‐hydroxypropionate since this species can consume the product (a common metabolic signature of many *Pseudomonas* species). Inactivation of the uptake system for 3‐hydroxypropionate in *P. denitrificans* appears to be a straightforward strategy to advancing the production of this valuable chemical in *Pseudomonas* – an approach that has been also adopted for the production of butanol in *P*. *putida* (Cuenca *et* *al.,* 2016).

## Conclusions and outlook

Over the last few years, glycerol has become an appealing choice for bioproduction, especially in processes designed for the synthesis of reduced chemicals. *Pseudomonas* species display a unique combination of genetic and metabolic architectures when growing on glycerol as the main carbon substrate, in particular, *P*. *putida* and *P*. *aeruginosa*, where the issue has been examined to some extent. As indicated in the first part of this article, multi‐omic strategies have strongly helped to elucidate the regulatory networks that rule glycerol utilization in *P*. *putida* KT2440 (including stochastic activation of genes encoding key enzymes needed for glycerol processing). *In silico*‐guided metabolic engineering strategies have also been implemented to increase the production of PHAs from this substrate. Admittedly, the full potential of glycerol as a biotechnological substrate for *Pseudomonas* has not been fully realized yet, but promising avenues can be envisioned in the near future – including novel strategies merging synthetic biology designs and laboratory evolution of engineered strains (Nørholm, 2019). First, once regulatory constraints for expression are overcome (e.g. by eliminating the GlpR repressor, as discussed above), core metabolic reactions linked to glycerol can be manipulated to foster synthesis of value‐added C3 compounds. For example, the connection of glycerol to biomass formation could be severed, and enzymatic sub‐networks could be set up for generating molecules of biotechnological interest, e.g. DHAP and derivatives thereof in resting cells [or, in any case, uncoupled from growth (Durante‐Rodríguez *et* *al.,* 2018; Volke *et* *al.,* 2019)]. Along the same lines, glycerol metabolism could be refactored to reduce carbon loss as CO_2_, while either concomitantly or separately, adjusting the redox balance to provide a better intracellular environment for hosting transformations of interest on other substrates. Furthermore, several metabolic routes could be rewired for fuelling the EDEMP cycle bottom‐up by means of a synthetic C3 neogenesis, empowering NADPH overproduction from glycerol processing, particularly useful for biosynthesis of reduced bioproducts. To this end, genetic editing of the *Pseudomonas* metabolism will benefit from systems biology approaches for simulating and predicting the effects of given mutations on specific carbon fluxes and pathways (Cho and Palsson, 2009; Gray *et* *al.,* 2015; Galardini *et* *al.,* 2017).

A second, considerable challenge is the further adaptation to meet the composition of industrial‐grade crude glycerol from biodiesel production. Typically, the glycerol stream has a polyol content in the range of 30‐65% (wt/vol), with the rest of the stream being CH_3_OH, fatty acid methyl esters, free fatty acids and glycerides together with ashes (Hu *et* *al.,* 2012). The waste also has a high pH due to the residual KOH or NaOH carried on from upstream transesterification of oils and fats that generate biodiesel. Industrial‐grade glycerol, often available as a non‐homogeneous oily mixture, is obviously quite different to what one can have in the controlled and pure‐substrate conditions of a shaken‐flask cultivation in the laboratory. While pre‐treatment (i.e. purification) may help improving the physical characteristics of this carbon source, bacteria have to ultimately face a mixture of compounds – some of them toxic and others not easily metabolizable. This offers again an opportunity to genetically knock in heterologous traits for the whole‐cell catalyst to endure the stressful conditions imposed by the use of crude glycerol. The issue here includes both endurance to the toxic effect of the non‐glycerol compounds of the mixture and the introduction of additional pathways for degrading or even growing on the additional carbon sources present in the medium. Some partial successes using industrial glycerol waste in bioproduction have been reported (see Table 1), yet the room for improvement in this field is still considerable.

Glycerol‐based bioprocesses have to be run in bioreactors, with a very large liquid‐to‐biomass ratio and sterile culture media that, after the operation takes place, need to be processed for purification of the molecules of interest. This scenario makes the production of such compounds costly and only appealing when the price tag of the thereby‐generated chemical is sufficiently high. The third avenue for improving glycerol valorization is therefore (re)designing the industrial engineering part of the bioprocesses better, and easing the downstream operations for reducing fermentation costs. This challenge not only applied to fermentations using this particular substrate, but to Microbial Biotechnology as a whole (de Lorenzo and Couto, 2019). Yet, even marginal improvement in bioprocess performance can make a considerable difference in the choice of substrates for feeding industrial‐scale production. In each of these fronts, synthetic biology and metabolic engineering are bound to contribute to the overarching goals of sustainable production from renewable resources, zero waste, and circular management of feedstocks and products, in the frame of the so‐called *4th Industrial Revolution* (Schwab, 2017).

## Conflicts of interest

None declared.
